# Stomatitis Materna

**Published:** 1848-03

**Authors:** 


*Stomatitis Materna.—The sore mouth of nursing Women.*—The editor of the *Buffalo Medical Journal*, Dr. Flint, makes the following valuable remarks on a disease of which we have lately met with cases.

"What is the pathology of the affection? We regard it as dependent upon anæmia, induced by lactation. The blood becomes impoverished by the continued drain upon its nutritive elements to form the lacteal secretion, and this is one of the local consequences which occasionally follow. In all cases that have fallen under our observation patients present unequivocal evidences of the anœmic state, and in proportion as this state has been relieved by appropriate measures, the affection of the mouth has improved. Why this local disorder should ensue as a result of anœmia we cannot tell, more than we can explain why various other local disorders are attributable to the same morbid condition. Still less can we understand why this particular local disorder should be characteristic of the anæmia induced by lactation or pregnancy, and not often, if ever, occur as a consequence of anæmia induced by other causes. In other words the fact that this form of stomatitis is peculiar to nursing women, in the present condition of science, is inexplicable. Doubtless there exists some special connection between the disease and the kind of impoverishment which the blood undergoes in consequence of the withdrawal from this fluid of the elements which go to form the lacteal secretion; but our knowledge of chemicovital chemistry does not suffice to trace the circumstances which constitute this special connection.

This is by no means the only instance in which the mucous membrane takes on disease as a result of prior deterioration of the fluids. We may instance the stomatitis which occurs in the last stages of pulmo-tuberculosis, and other chronic diseases; softening of the mucous tissue of the stomach and intestines from inanition; scrofulous ophthalmia; perhaps we may add the follicular affection of typhoid fever, &c.

That the affection has seemed to arise from miasmata is explicable, when we consider that to the latter alone anæmia is often referable, and consequently the effect of lactation is increased by conjunction with miasmatic influences. That it is wholly due to miasm is, however, sufficiently disproved by the fact that it occurs in localities where miasmatic emanations are unknown.

If this view of the pathology be correct, the proper therapeutical principles are readily deducible. The seat of the disease is not in the mouth, but it is a blood disease. Hence, we should expect, what experience declares to be the case, that collutories are of little or no avail. To reach the *fons et origo* of the difficulty, the blood must be restored to a healthy condition.

The first question of the practitioner is, can this be accomplished while the remote cause of the disease (lactation) continues. Our own experience teaches us that in the large proportion of cases, weaning becomes necessary. This affection frequently does not occur until the blood has undergone deterioration to such extent that restoration is impossible without removing the remote cause. Whenever the patient is much prostrated, and, especially, if diarrhœa co-exists, the lacteal secretion should be allowed to cease without delay.

In most cases, however, it will be proper to give remedies a fair trial before deciding on the necessity of weaning. And, if we mistake not, the success of the treatment dictated by the foregoing view of its pathology, will, in not a few instances, strikingly exemplify the correctness of the latter. The treatment is that called for by the anæmic state, viz: nutritious diet, especially animal food and ferruginous tonics. Should the bowels be costive, or any functions be disordered, they will claim appropriate measures, but the grand object is to improve the powers of assimilation, and thereby restore the healthy condition of the blood. A great difficulty with a view to this object arises from the inability of the patient to take proper food in proper quantities, owing to the tenderness of the mouth. We have known patients who constantly suffer from hunger, which they would endure, rather than incur the pain incident to satisfying fully the appetite. Local applications should be tried, and, by alleviating the soreness, they may conduce to permanent benefit, by permitting the patient to take more nourishment. To the local remedies which are mentioned in the foregoing article, we would add, a solution of tannin in port wine, used as a collutory, of sufficient strength to cause a decidedly astringent effect, without occasioning much pain. We have employed this remedy, of late, in some cases, with advantage.

Of the tonics to be prescribed, there are various preparations which are appropriate, and the peculiarities of different cases will probably be suited by different forms. The citrate of iron, in combination with quinia, given in concentrated solution, as suggested by Dr. Meigs, has been a favorite prescription with us, and we know of no form more generally applicable.

Exercise in the open air appears to us one of the most important elements in the treatment.

Frequently the child may be partially weaned. Some mothers are accustomed to nurse frequently, or almost constantly during the night time, when loss of sleep is to be added to the cause of the disease which is more direct in its operation. The foolish custom should be interdicted.

Should these measures fail, weaning should be directed, and, in nearly all cases, the disease rapidly subsides so soon as this is done.

